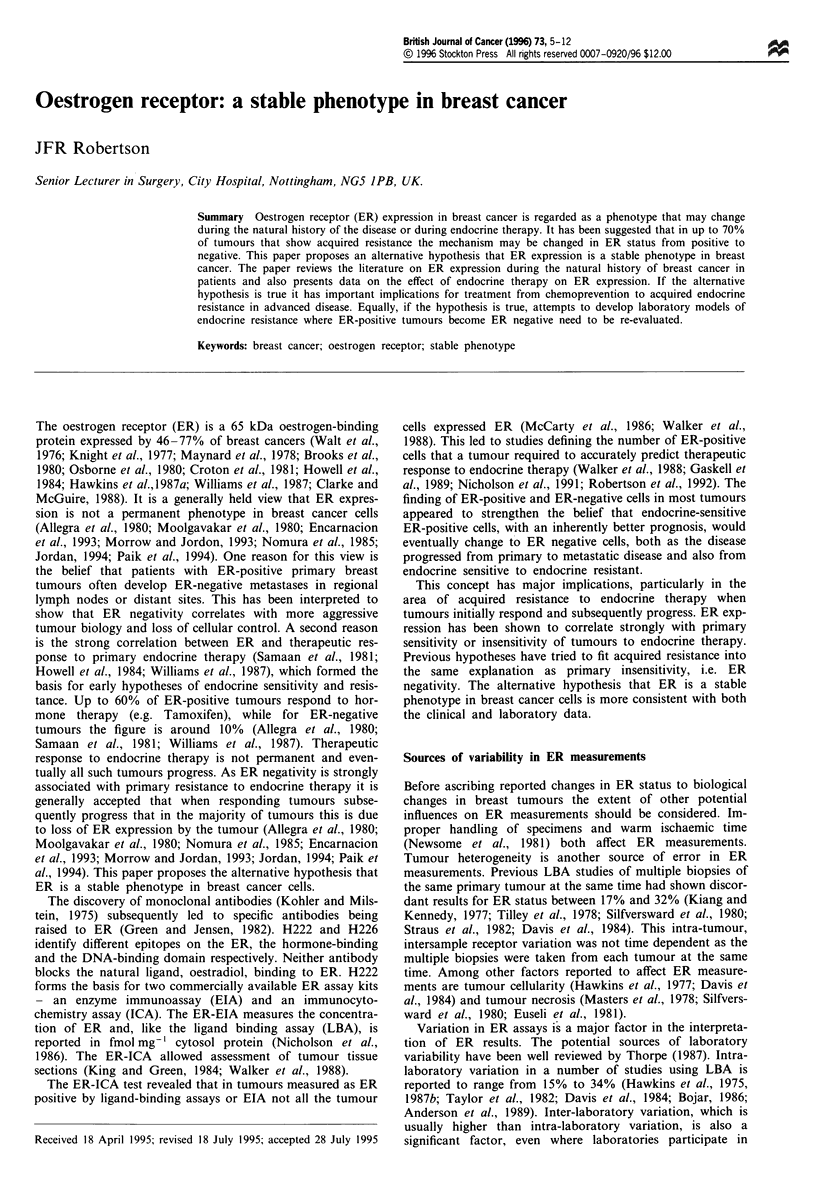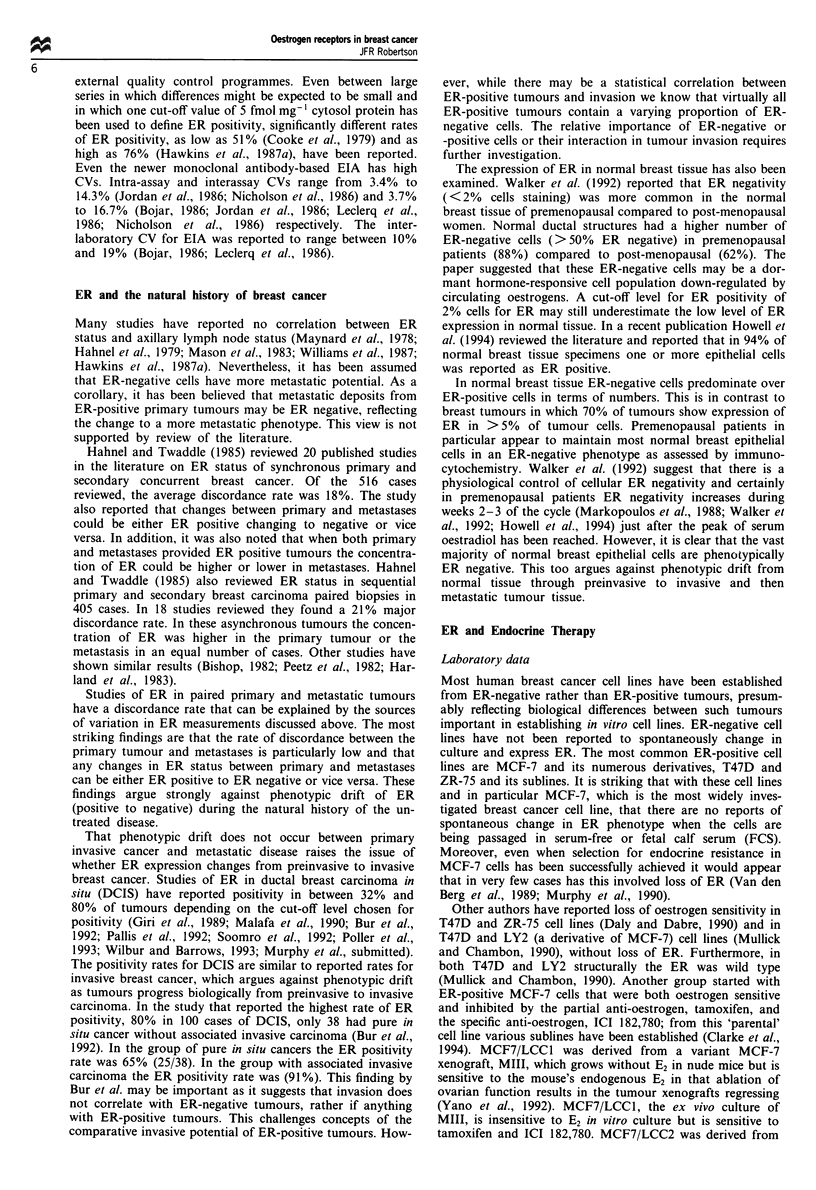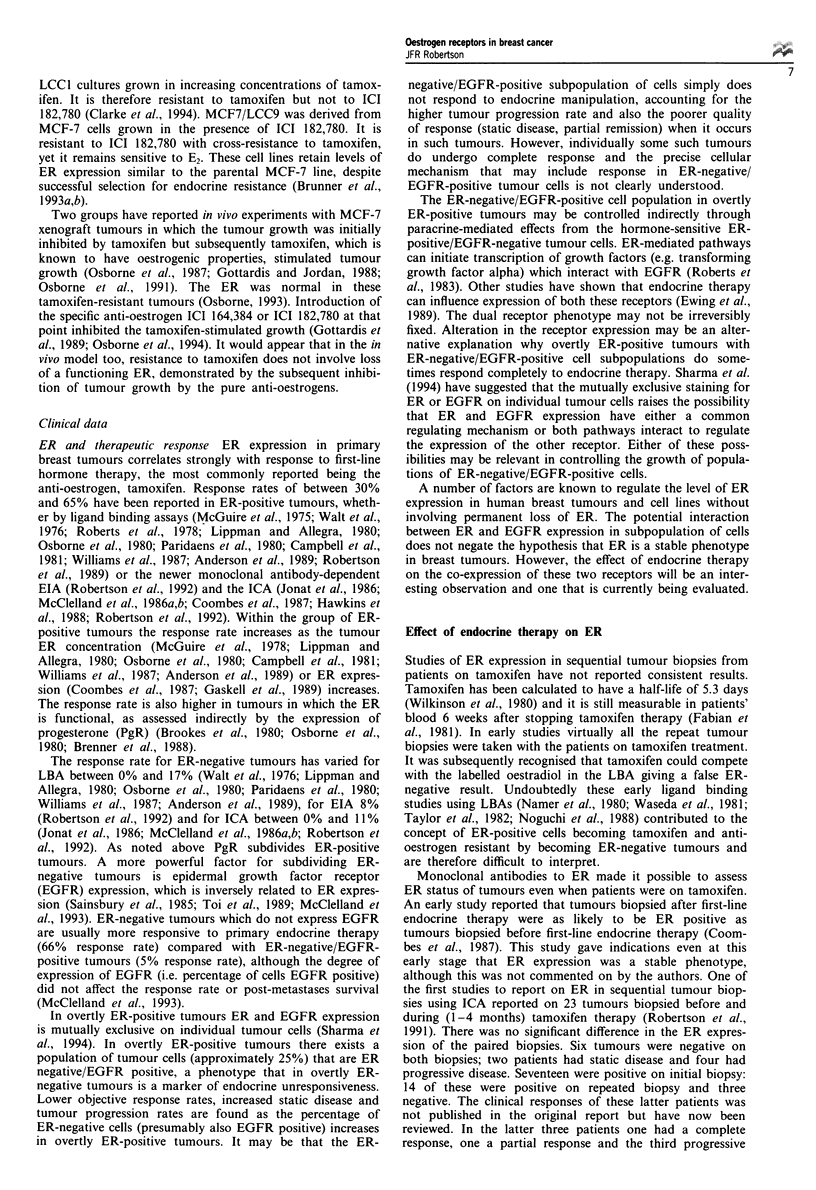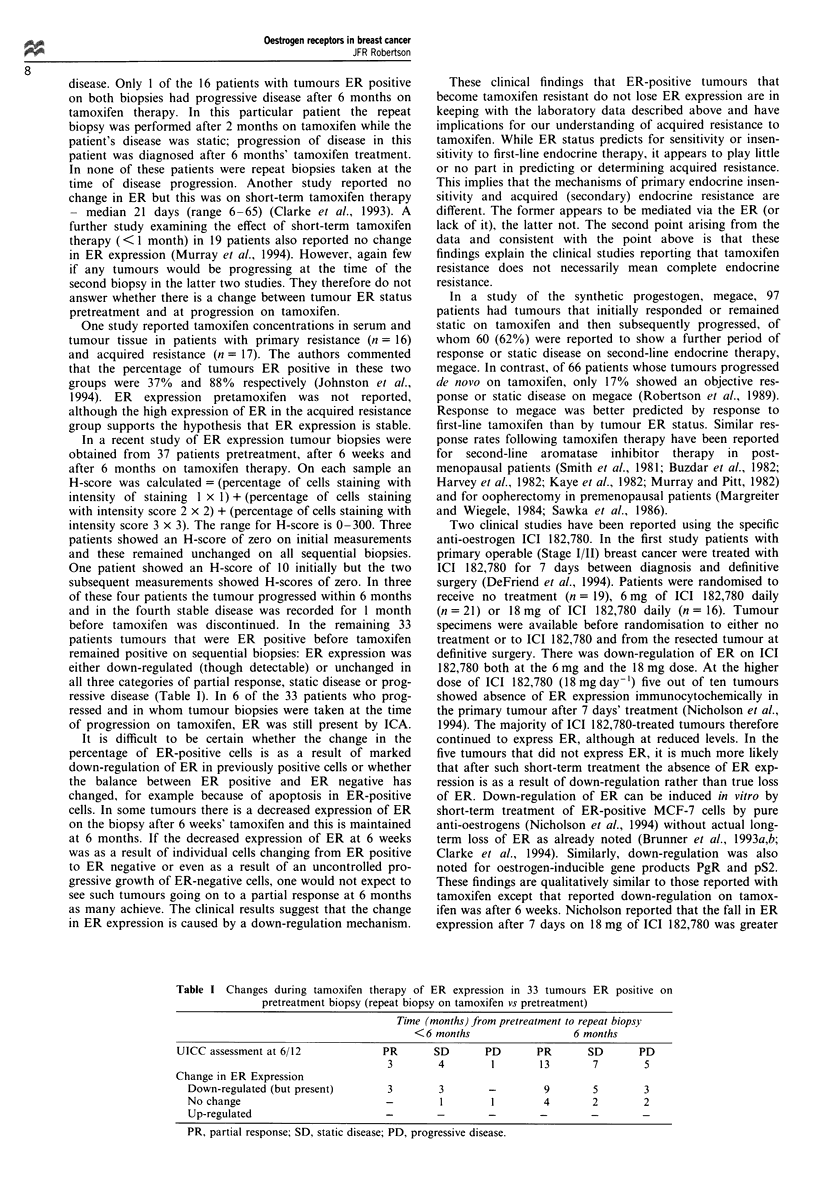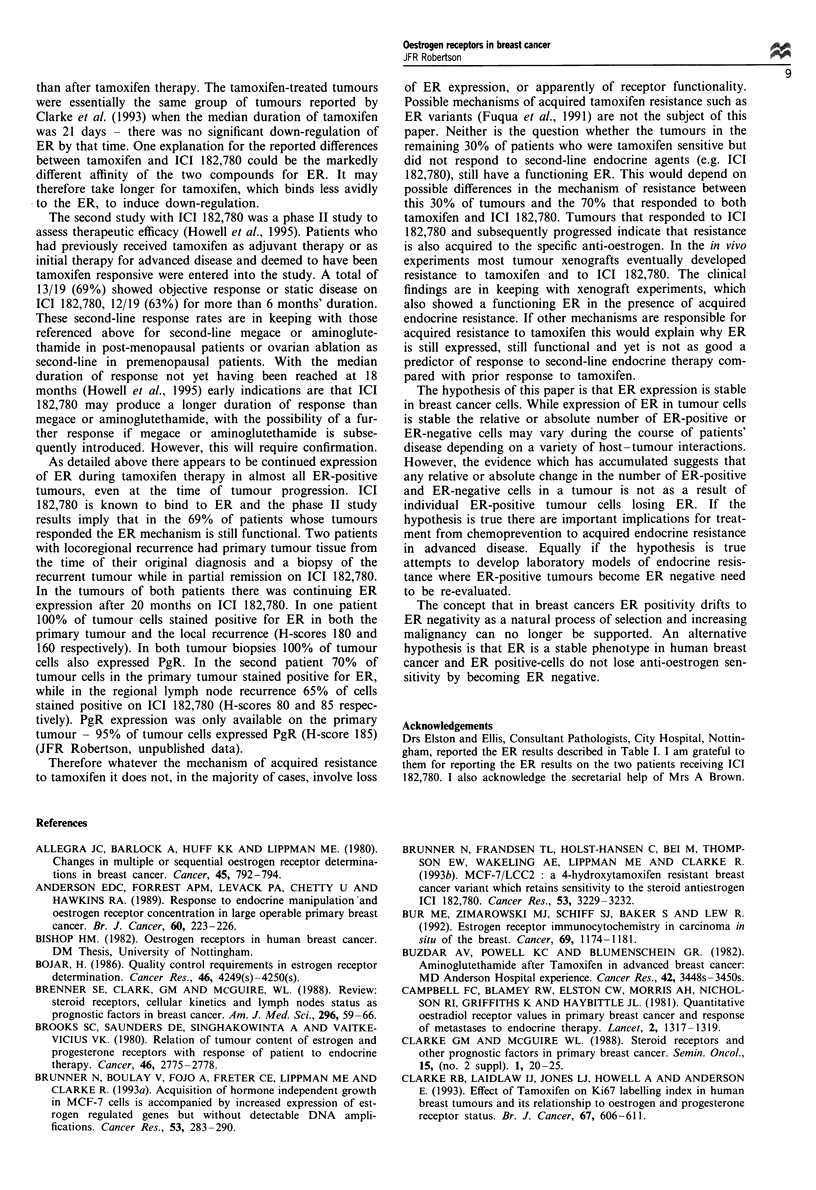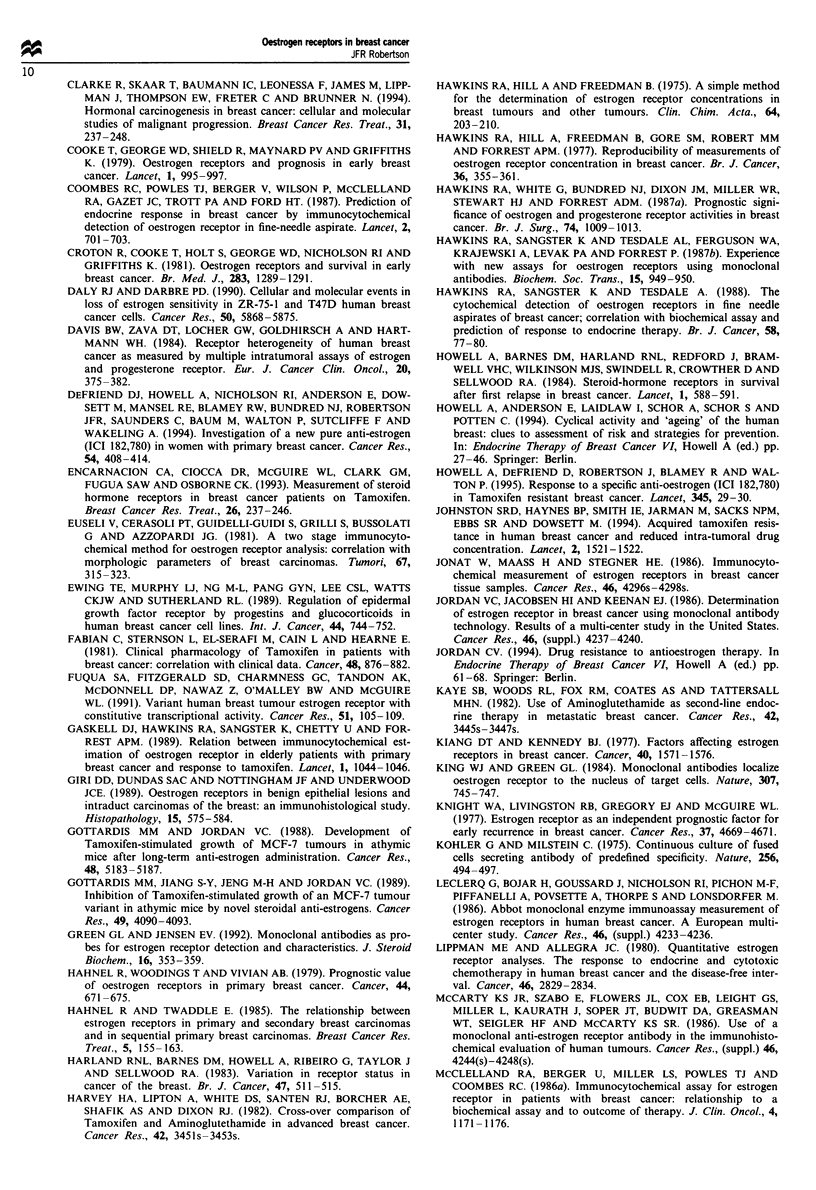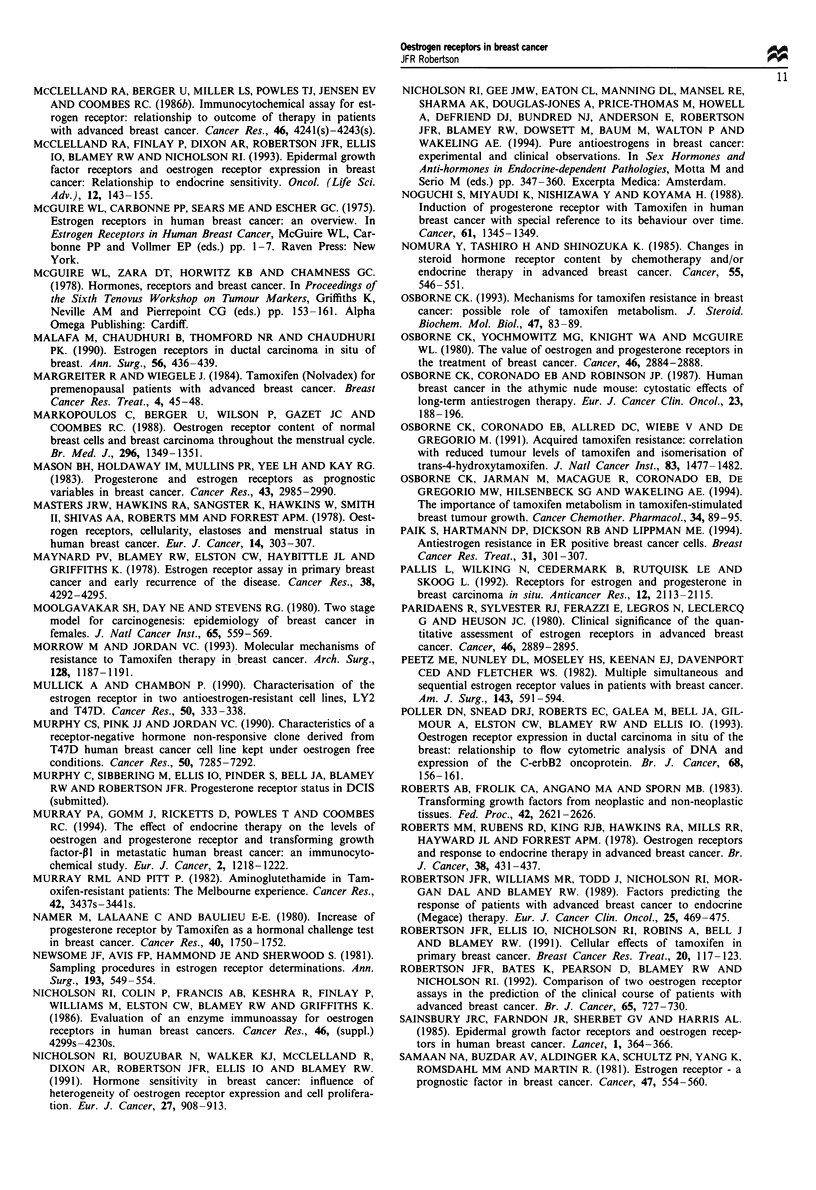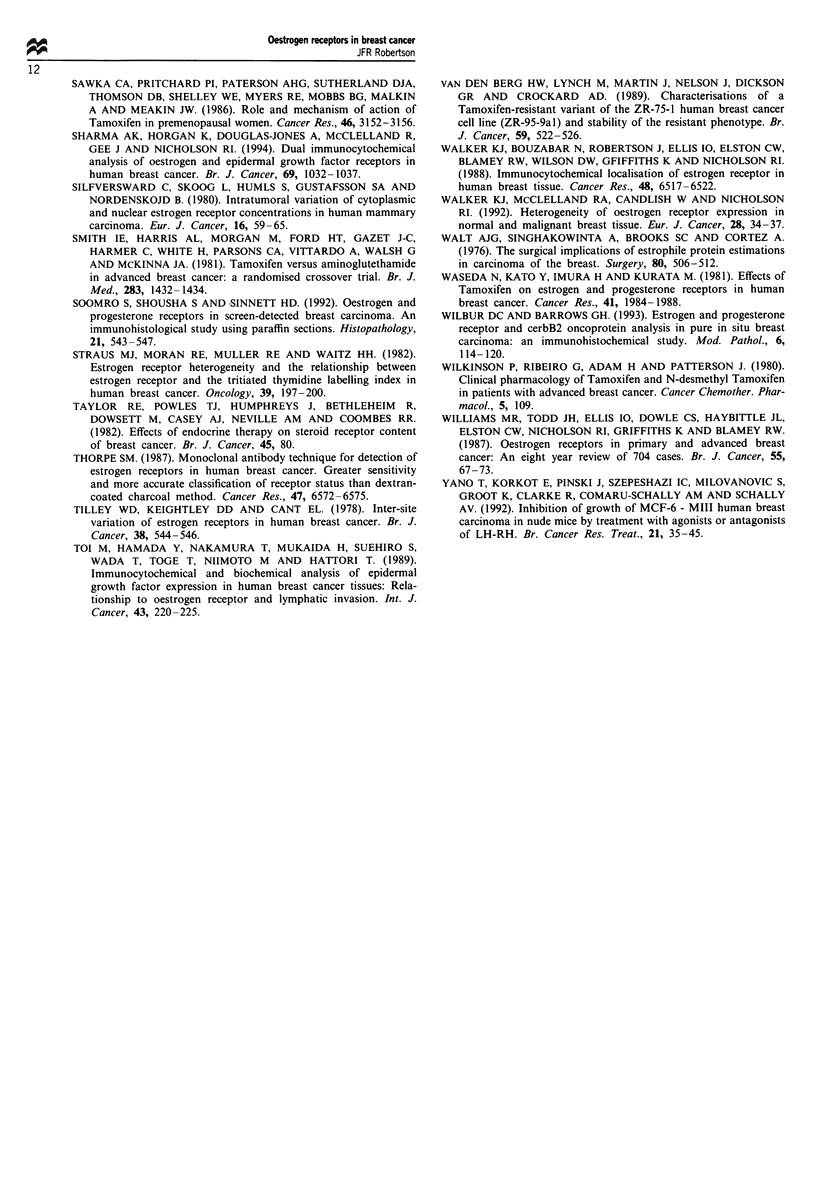# Oestrogen receptor: a stable phenotype in breast cancer.

**DOI:** 10.1038/bjc.1996.2

**Published:** 1996-01

**Authors:** J. F. Robertson

**Affiliations:** City Hospital, Nottingham, UK.

## Abstract

Oestrogen receptor (ER) expression in breast cancer is regarded as a phenotype that may change during the natural history of the disease or during endocrine therapy. It has been suggested that in up to 70% of tumours that show acquired resistance the mechanism may be changed in ER status from positive to negative. This paper proposes an alternative hypothesis that ER expression in a stable phenotype in breast cancer. The paper reviews the literature on ER expression during the natural history of breast cancer in patients and also presents data on the effect of endocrine therapy on ER expression. If the alternative hypothesis is true it has important implications for treatment from chemoprevention to acquired endocrine resistance in advanced disease. Equally, if the hypothesis is true, attempts to develop laboratory models of endocrine resistance where ER-positive tumours become ER negative need to be re-evaluated.


British Journal of Cancer (1996) 73, 5-12
© 1996 Stockton Press All rights reserved 0007-0920/96 $12.00

### Oestrogen receptor: a stable phenotype in breast cancer

JFR Robertson

*Senior Lecturer in Surgery, City Hospital, Nottingham, NG5 1PB, UK.*

**Summary** Oestrogen receptor (ER) expression in breast cancer is regarded as a phenotype that may change during the natural history of the disease or during endocrine therapy. It has been suggested that in up to 70% of tumours that show acquired resistance the mechanism may be changed in ER status from positive to negative. This paper proposes an alternative hypothesis that ER expression is a stable phenotype in breast cancer. The paper reviews the literature on ER expression during the natural history of breast cancer in patients and also presents data on the effect of endocrine therapy on ER expression. If the alternative hypothesis is true it has important implications for treatment from chemoprevention to acquired endocrine resistance in advanced disease. Equally, if the hypothesis is true, attempts to develop laboratory models of endocrine resistance where ER-positive tumours become ER negative need to be re-evaluated.

**Keywords:** breast cancer; oestrogen receptor; stable phenotype

The oestrogen receptor (ER) is a 65 kDa oestrogen-binding protein expressed by 46–77% of breast cancers (Walt *et al.*, 1976; Knight *et al.*, 1977; Maynard *et al.*, 1978; Brooks *et al.*, 1980; Osborne *et al.*, 1980; Croton *et al.*, 1981; Howell *et al.*, 1984; Hawkins *et al.*,1987*a*; Williams *et al.*, 1987; Clarke and McGuire, 1988). It is a generally held view that ER expression is not a permanent phenotype in breast cancer cells (Allegra *et al.*, 1980; Moolgavakar *et al.*, 1980; Encarnacion *et al.*, 1993; Morrow and Jordon, 1993; Nomura *et al.*, 1985; Jordan, 1994; Paik *et al.*, 1994). One reason for this view is the belief that patients with ER-positive primary breast tumours often develop ER-negative metastases in regional lymph nodes or distant sites. This has been interpreted to show that ER negativity correlates with more aggressive tumour biology and loss of cellular control. A second reason is the strong correlation between ER and therapeutic response to primary endocrine therapy (Samaan *et al.*, 1981; Howell *et al.*, 1984; Williams *et al.*, 1987), which formed the basis for early hypotheses of endocrine sensitivity and resistance. Up to 60% of ER-positive tumours respond to hormone therapy (e.g. Tamoxifen), while for ER-negative tumours the figure is around 10% (Allegra *et al.*, 1980; Samaan *et al.*, 1981; Williams *et al.*, 1987). Therapeutic response to endocrine therapy is not permanent and eventually all such tumours progress. As ER negativity is strongly associated with primary resistance to endocrine therapy it is generally accepted that when responding tumours subsequently progress that in the majority of tumours this is due to loss of ER expression by the tumour (Allegra *et al.*, 1980; Moolgavakar *et al.*, 1980; Nomura *et al.*, 1985; Encarnacion *et al.*, 1993; Morrow and Jordan, 1993; Jordan, 1994; Paik *et al.*, 1994). This paper proposes the alternative hypothesis that ER is a stable phenotype in breast cancer cells.

The discovery of monoclonal antibodies (Kohler and Milstein, 1975) subsequently led to specific antibodies being raised to ER (Green and Jensen, 1982). H222 and H226 identify different epitopes on the ER, the hormone-binding and the DNA-binding domain respectively. Neither antibody blocks the natural ligand, oestradiol, binding to ER. H222 forms the basis for two commercially available ER assay kits – an enzyme immunoassay (EIA) and an immunocytochemistry assay (ICA). The ER-EIA measures the concentration of ER and, like the ligand binding assay (LBA), is reported in fmol $mg^{-1}$ cytosol protein (Nicholson *et al.*, 1986). The ER-ICA allowed assessment of tumour tissue sections (King and Green, 1984; Walker *et al.*, 1988).

The ER-ICA test revealed that in tumours measured as ER positive by ligand-binding assays or EIA not all the tumour cells expressed ER (McCarty *et al.*, 1986; Walker *et al.*, 1988). This led to studies defining the number of ER-positive cells that a tumour required to accurately predict therapeutic response to endocrine therapy (Walker *et al.*, 1988; Gaskell *et al.*, 1989; Nicholson *et al.*, 1991; Robertson *et al.*, 1992). The finding of ER-positive and ER-negative cells in most tumours appeared to strengthen the belief that endocrine-sensitive ER-positive cells, with an inherently better prognosis, would eventually change to ER negative cells, both as the disease progressed from primary to metastatic disease and also from endocrine sensitive to endocrine resistant.

This concept has major implications, particularly in the area of acquired resistance to endocrine therapy when tumours initially respond and subsequently progress. ER expression has been shown to correlate strongly with primary sensitivity or insensitivity of tumours to endocrine therapy. Previous hypotheses have tried to fit acquired resistance into the same explanation as primary insensitivity, i.e. ER negativity. The alternative hypothesis that ER is a stable phenotype in breast cancer cells is more consistent with both the clinical and laboratory data.

### Sources of variability in ER measurements

Before ascribing reported changes in ER status to biological changes in breast tumours the extent of other potential influences on ER measurements should be considered. Improper handling of specimens and warm ischaemic time (Newsome *et al.*, 1981) both affect ER measurements. Tumour heterogeneity is another source of error in ER measurements. Previous LBA studies of multiple biopsies of the same primary tumour at the same time had shown discordant results for ER status between 17% and 32% (Kiang and Kennedy, 1977; Tilley *et al.*, 1978; Silfversward *et al.*, 1980; Straus *et al.*, 1982; Davis *et al.*, 1984). This intra-tumour, intersample receptor variation was not time dependent as the multiple biopsies were taken from each tumour at the same time. Among other factors reported to affect ER measurements are tumour cellularity (Hawkins *et al.*, 1977; Davis *et al.*, 1984) and tumour necrosis (Masters *et al.*, 1978; Silfversward *et al.*, 1980; Euseli *et al.*, 1981).

Variation in ER assays is a major factor in the interpretation of ER results. The potential sources of laboratory variability have been well reviewed by Thorpe (1987). Intra-laboratory variation in a number of studies using LBA is reported to range from 15% to 34% (Hawkins *et al.*, 1975, 1987*b*; Taylor *et al.*, 1982; Davis *et al.*, 1984; Bojar, 1986; Anderson *et al.*, 1989). Inter-laboratory variation, which is usually higher than intra-laboratory variation, is also a significant factor, even where laboratories participate in

Received 18 April 1995; revised 18 July 1995; accepted 28 July 1995

external quality control programmes. Even between large series in which differences might be expected to be small and in which one cut-off value of 5 fmol $mg^{-1}$ cytosol protein has been used to define ER positivity, significantly different rates of ER positivity, as low as 51% (Cooke *et al.*, 1979) and as high as 76% (Hawkins *et al.*, 1987*a*), have been reported. Even the newer monoclonal antibody-based EIA has high CVs. Intra-assay and interassay CVs range from 3.4% to 14.3% (Jordan *et al.*, 1986; Nicholson *et al.*, 1986) and 3.7% to 16.7% (Bojar, 1986; Jordan *et al.*, 1986; Leclerq *et al.*, 1986; Nicholson *et al.*, 1986) respectively. The inter-laboratory CV for EIA was reported to range between 10% and 19% (Bojar, 1986; Leclerq *et al.*, 1986).

### ER and the natural history of breast cancer

Many studies have reported no correlation between ER status and axillary lymph node status (Maynard *et al.*, 1978; Hahnel *et al.*, 1979; Mason *et al.*, 1983; Williams *et al.*, 1987; Hawkins *et al.*, 1987*a*). Nevertheless, it has been assumed that ER-negative cells have more metastatic potential. As a corollary, it has been believed that metastatic deposits from ER-positive primary tumours may be ER negative, reflecting the change to a more metastatic phenotype. This view is not supported by review of the literature.

Hahnel and Twaddle (1985) reviewed 20 published studies in the literature on ER status of synchronous primary and secondary concurrent breast cancer. Of the 516 cases reviewed, the average discordance rate was 18%. The study also reported that changes between primary and metastases could be either ER positive changing to negative or vice versa. In addition, it was also noted that when both primary and metastases provided ER positive tumours the concentration of ER could be higher or lower in metastases. Hahnel and Twaddle (1985) also reviewed ER status in sequential primary and secondary breast carcinoma paired biopsies in 405 cases. In 18 studies reviewed they found a 21% major discordance rate. In these asynchronous tumours the concentration of ER was higher in the primary tumour or the metastasis in an equal number of cases. Other studies have shown similar results (Bishop, 1982; Peetz *et al.*, 1982; Harland *et al.*, 1983).

Studies of ER in paired primary and metastatic tumours have a discordance rate that can be explained by the sources of variation in ER measurements discussed above. The most striking findings are that the rate of discordance between the primary tumour and metastases is particularly low and that any changes in ER status between primary and metastases can be either ER positive to ER negative or vice versa. These findings argue strongly against phenotypic drift of ER (positive to negative) during the natural history of the untreated disease.

That phenotypic drift does not occur between primary invasive cancer and metastatic disease raises the issue of whether ER expression changes from preinvasive to invasive breast cancer. Studies of ER in ductal breast carcinoma *in situ* (DCIS) have reported positivity in between 32% and 80% of tumours depending on the cut-off level chosen for positivity (Giri *et al.*, 1989; Malafa *et al.*, 1990; Bur *et al.*, 1992; Pallis *et al.*, 1992; Soomro *et al.*, 1992; Poller *et al.*, 1993; Wilbur and Barrows, 1993; Murphy *et al.*, submitted). The positivity rates for DCIS are similar to reported rates for invasive breast cancer, which argues against phenotypic drift as tumours progress biologically from preinvasive to invasive carcinoma. In the study that reported the highest rate of ER positivity, 80% in 100 cases of DCIS, only 38 had pure *in situ* cancer without associated invasive carcinoma (Bur *et al.*, 1992). In the group of pure *in situ* cancers the ER positivity rate was 65% (25/38). In the group with associated invasive carcinoma the ER positivity rate was (91%). This finding by Bur *et al.* may be important as it suggests that invasion does not correlate with ER-negative tumours, rather if anything with ER-positive tumours. This challenges concepts of the comparative invasive potential of ER-positive tumours. However, while there may be a statistical correlation between ER-positive tumours and invasion we know that virtually all ER-positive tumours contain a varying proportion of ER-negative cells. The relative importance of ER-negative or -positive cells or their interaction in tumour invasion requires further investigation.

The expression of ER in normal breast tissue has also been examined. Walker *et al.* (1992) reported that ER negativity (<2% cells staining) was more common in the normal breast tissue of premenopausal compared to post-menopausal women. Normal ductal structures had a higher number of ER-negative cells (>50% ER negative) in premenopausal patients (88%) compared to post-menopausal (62%). The paper suggested that these ER-negative cells may be a dormant hormone-responsive cell population down-regulated by circulating oestrogens. A cut-off level for ER positivity of 2% cells for ER may still underestimate the low level of ER expression in normal tissue. In a recent publication Howell *et al.* (1994) reviewed the literature and reported that in 94% of normal breast tissue specimens one or more epithelial cells was reported as ER positive.

In normal breast tissue ER-negative cells predominate over ER-positive cells in terms of numbers. This is in contrast to breast tumours in which 70% of tumours show expression of ER in >5% of tumour cells. Premenopausal patients in particular appear to maintain most normal breast epithelial cells in an ER-negative phenotype as assessed by immunocytochemistry. Walker *et al.* (1992) suggest that there is a physiological control of cellular ER negativity and certainly in premenopausal patients ER negativity increases during weeks 2–3 of the cycle (Markopoulos *et al.*, 1988; Walker *et al.*, 1992; Howell *et al.*, 1994) just after the peak of serum oestradiol has been reached. However, it is clear that the vast majority of normal breast epithelial cells are phenotypically ER negative. This too argues against phenotypic drift from normal tissue through preinvasive to invasive and then metastatic tumour tissue.

### ER and Endocrine Therapy

### *Laboratory data*

Most human breast cancer cell lines have been established from ER-negative rather than ER-positive tumours, presumably reflecting biological differences between such tumours important in establishing *in vitro* cell lines. ER-negative cell lines have not been reported to spontaneously change in culture and express ER. The most common ER-positive cell lines are MCF-7 and its numerous derivatives, T47D and ZR-75 and its sublines. It is striking that with these cell lines and in particular MCF-7, which is the most widely investigated breast cancer cell line, that there are no reports of spontaneous change in ER phenotype when the cells are being passaged in serum-free or fetal calf serum (FCS). Moreover, even when selection for endocrine resistance in MCF-7 cells has been successfully achieved it would appear that in very few cases has this involved loss of ER (Van den Berg *et al.*, 1989; Murphy *et al.*, 1990).

Other authors have reported loss of oestrogen sensitivity in T47D and ZR-75 cell lines (Daly and Dabre, 1990) and in T47D and LY2 (a derivative of MCF-7) cell lines (Mullick and Chambon, 1990), without loss of ER. Furthermore, in both T47D and LY2 structurally the ER was wild type (Mullick and Chambon, 1990). Another group started with ER-positive MCF-7 cells that were both oestrogen sensitive and inhibited by the partial anti-oestrogen, tamoxifen, and the specific anti-oestrogen, ICI 182,780; from this 'parental' cell line various sublines have been established (Clarke *et al.*, 1994). MCF7/LCC1 was derived from a variant MCF-7 xenograft, MIII, which grows without $E_2$ in nude mice but is sensitive to the mouse's endogenous $E_2$ in that ablation of ovarian function results in the tumour xenografts regressing (Yano *et al.*, 1992). MCF7/LCC1, the *ex vivo* culture of MIII, is insensitive to $E_2$ *in vitro* culture but is sensitive to tamoxifen and ICI 182,780. MCF7/LCC2 was derived from

LCC1 cultures grown in increasing concentrations of tamoxifen. It is therefore resistant to tamoxifen but not to ICI 182,780 (Clarke *et al.*, 1994). MCF7/LCC9 was derived from MCF-7 cells grown in the presence of ICI 182,780. It is resistant to ICI 182,780 with cross-resistance to tamoxifen, yet it remains sensitive to $E_2$. These cell lines retain levels of ER expression similar to the parental MCF-7 line, despite successful selection for endocrine resistance (Brunner *et al.*, 1993*a,b*).

Two groups have reported *in vivo* experiments with MCF-7 xenograft tumours in which the tumour growth was initially inhibited by tamoxifen but subsequently tamoxifen, which is known to have oestrogenic properties, stimulated tumour growth (Osborne *et al.*, 1987; Gottardis and Jordan, 1988; Osborne *et al.*, 1991). The ER was normal in these tamoxifen-resistant tumours (Osborne, 1993). Introduction of the specific anti-oestrogen ICI 164,384 or ICI 182,780 at that point inhibited the tamoxifen-stimulated growth (Gottardis *et al.*, 1989; Osborne *et al.*, 1994). It would appear that in the *in vivo* model too, resistance to tamoxifen does not involve loss of a functioning ER, demonstrated by the subsequent inhibition of tumour growth by the pure anti-oestrogens.

### *Clinical data*

*ER and therapeutic response* ER expression in primary breast tumours correlates strongly with response to first-line hormone therapy, the most commonly reported being the anti-oestrogen, tamoxifen. Response rates of between 30% and 65% have been reported in ER-positive tumours, whether by ligand binding assays (McGuire *et al.*, 1975; Walt *et al.*, 1976; Roberts *et al.*, 1978; Lippman and Allegra, 1980; Osborne *et al.*, 1980; Paridaens *et al.*, 1980; Campbell *et al.*, 1981; Williams *et al.*, 1987; Anderson *et al.*, 1989; Robertson *et al.*, 1989) or the newer monoclonal antibody-dependent EIA (Robertson *et al.*, 1992) and the ICA (Jonat *et al.*, 1986; McClelland *et al.*, 1986*a,b*; Coombes *et al.*, 1987; Hawkins *et al.*, 1988; Robertson *et al.*, 1992). Within the group of ER-positive tumours the response rate increases as the tumour ER concentration (McGuire *et al.*, 1978; Lippman and Allegra, 1980; Osborne *et al.*, 1980; Campbell *et al.*, 1981; Williams *et al.*, 1987; Anderson *et al.*, 1989) or ER expression (Coombes *et al.*, 1987; Gaskell *et al.*, 1989) increases. The response rate is also higher in tumours in which the ER is functional, as assessed indirectly by the expression of progesterone (PgR) (Brookes *et al.*, 1980; Osborne *et al.*, 1980; Brenner *et al.*, 1988).

The response rate for ER-negative tumours has varied for LBA between 0% and 17% (Walt *et al.*, 1976; Lippman and Allegra, 1980; Osborne *et al.*, 1980; Paridaens *et al.*, 1980; Williams *et al.*, 1987; Anderson *et al.*, 1989), for EIA 8% (Robertson *et al.*, 1992) and for ICA between 0% and 11% (Jonat *et al.*, 1986; McClelland *et al.*, 1986*a,b*; Robertson *et al.*, 1992). As noted above PgR subdivides ER-positive tumours. A more powerful factor for subdividing ER-negative tumours is epidermal growth factor receptor (EGFR) expression, which is inversely related to ER expression (Sainsbury *et al.*, 1985; Toi *et al.*, 1989; McClelland *et al.*, 1993). ER-negative tumours which do not express EGFR are usually more responsive to primary endocrine therapy (66% response rate) compared with ER-negative/EGFR-positive tumours (5% response rate), although the degree of expression of EGFR (i.e. percentage of cells EGFR positive) did not affect the response rate or post-metastases survival (McClelland *et al.*, 1993).

In overtly ER-positive tumours ER and EGFR expression is mutually exclusive on individual tumour cells (Sharma *et al.*, 1994). In overtly ER-positive tumours there exists a population of tumour cells (approximately 25%) that are ER negative/EGFR positive, a phenotype that in overtly ER-negative tumours is a marker of endocrine unresponsiveness. Lower objective response rates, increased static disease and tumour progression rates are found as the percentage of ER-negative cells (presumably also EGFR positive) increases in overtly ER-positive tumours. It may be that the ER-negative/EGFR-positive subpopulation of cells simply does not respond to endocrine manipulation, accounting for the higher tumour progression rate and also the poorer quality of response (static disease, partial remission) when it occurs in such tumours. However, individually some such tumours do undergo complete response and the precise cellular mechanism that may include response in ER-negative/EGFR-positive tumour cells is not clearly understood.

The ER-negative/EGFR-positive cell population in overtly ER-positive tumours may be controlled indirectly through paracrine-mediated effects from the hormone-sensitive ER-positive/EGFR-negative tumour cells. ER-mediated pathways can initiate transcription of growth factors (e.g. transforming growth factor alpha) which interact with EGFR (Roberts *et al.*, 1983). Other studies have shown that endocrine therapy can influence expression of both these receptors (Ewing *et al.*, 1989). The dual receptor phenotype may not be irreversibly fixed. Alteration in the receptor expression may be an alternative explanation why overtly ER-positive tumours with ER-negative/EGFR-positive cell subpopulations do sometimes respond completely to endocrine therapy. Sharma *et al.* (1994) have suggested that the mutually exclusive staining for ER or EGFR on individual tumour cells raises the possibility that ER and EGFR expression have either a common regulating mechanism or both pathways interact to regulate the expression of the other receptor. Either of these possibilities may be relevant in controlling the growth of populations of ER-negative/EGFR-positive cells.

A number of factors are known to regulate the level of ER expression in human breast tumours and cell lines without involving permanent loss of ER. The potential interaction between ER and EGFR expression in subpopulation of cells does not negate the hypothesis that ER is a stable phenotype in breast tumours. However, the effect of endocrine therapy on the co-expression of these two receptors will be an interesting observation and one that is currently being evaluated.

### Effect of endocrine therapy on ER

Studies of ER expression in sequential tumour biopsies from patients on tamoxifen have not reported consistent results. Tamoxifen has been calculated to have a half-life of 5.3 days (Wilkinson *et al.*, 1980) and it is still measurable in patients' blood 6 weeks after stopping tamoxifen therapy (Fabian *et al.*, 1981). In early studies virtually all the repeat tumour biopsies were taken with the patients on tamoxifen treatment. It was subsequently recognised that tamoxifen could compete with the labelled oestradiol in the LBA giving a false ER-negative result. Undoubtedly these early ligand binding studies using LBAs (Namer *et al.*, 1980; Waseda *et al.*, 1981; Taylor *et al.*, 1982; Noguchi *et al.*, 1988) contributed to the concept of ER-positive cells becoming tamoxifen and anti-oestrogen resistant by becoming ER-negative tumours and are therefore difficult to interpret.

Monoclonal antibodies to ER made it possible to assess ER status of tumours even when patients were on tamoxifen. An early study reported that tumours biopsied after first-line endocrine therapy were as likely to be ER positive as tumours biopsied before first-line endocrine therapy (Coombes *et al.*, 1987). This study gave indications even at this early stage that ER expression was a stable phenotype, although this was not commented on by the authors. One of the first studies to report on ER in sequential tumour biopsies using ICA reported on 23 tumours biopsied before and during (1–4 months) tamoxifen therapy (Robertson *et al.*, 1991). There was no significant difference in the ER expression of the paired biopsies. Six tumours were negative on both biopsies; two patients had static disease and four had progressive disease. Seventeen were positive on initial biopsy: 14 of these were positive on repeated biopsy and three negative. The clinical responses of these latter patients was not published in the original report but have now been reviewed. In the latter three patients one had a complete response, one a partial response and the third progressive disease. Only 1 of the 16 patients with tumours ER positive on both biopsies had progressive disease after 6 months on tamoxifen therapy. In this particular patient the repeat biopsy was performed after 2 months on tamoxifen while the patient's disease was static; progression of disease in this patient was diagnosed after 6 months' tamoxifen treatment. In none of these patients were repeat biopsies taken at the time of disease progression. Another study reported no change in ER but this was on short-term tamoxifen therapy – median 21 days (range 6–65) (Clarke *et al.*, 1993). A further study examining the effect of short-term tamoxifen therapy ($<1$ month) in 19 patients also reported no change in ER expression (Murray *et al.*, 1994). However, again few if any tumours would be progressing at the time of the second biopsy in the latter two studies. They therefore do not answer whether there is a change between tumour ER status pretreatment and at progression on tamoxifen.

One study reported tamoxifen concentrations in serum and tumour tissue in patients with primary resistance ($n = 16$) and acquired resistance ($n = 17$). The authors commented that the percentage of tumours ER positive in these two groups were 37% and 88% respectively (Johnston *et al.*, 1994). ER expression pretamoxifen was not reported, although the high expression of ER in the acquired resistance group supports the hypothesis that ER expression is stable.

In a recent study of ER expression tumour biopsies were obtained from 37 patients pretreatment, after 6 weeks and after 6 months on tamoxifen therapy. On each sample an H-score was calculated = (percentage of cells staining with intensity of staining $1 \times 1$) + (percentage of cells staining with intensity score $2 \times 2$) + (percentage of cells staining with intensity score $3 \times 3$). The range for H-score is 0–300. Three patients showed an H-score of zero on initial measurements and these remained unchanged on all sequential biopsies. One patient showed an H-score of 10 initially but the two subsequent measurements showed H-scores of zero. In three of these four patients the tumour progressed within 6 months and in the fourth stable disease was recorded for 1 month before tamoxifen was discontinued. In the remaining 33 patients tumours that were ER positive before tamoxifen remained positive on sequential biopsies: ER expression was either down-regulated (though detectable) or unchanged in all three categories of partial response, static disease or progressive disease (Table I). In 6 of the 33 patients who progressed and in whom tumour biopsies were taken at the time of progression on tamoxifen, ER was still present by ICA.

It is difficult to be certain whether the change in the percentage of ER-positive cells is as a result of marked down-regulation of ER in previously positive cells or whether the balance between ER positive and ER negative has changed, for example because of apoptosis in ER-positive cells. In some tumours there is a decreased expression of ER on the biopsy after 6 weeks' tamoxifen and this is maintained at 6 months. If the decreased expression of ER at 6 weeks was as a result of individual cells changing from ER positive to ER negative or even as a result of an uncontrolled progressive growth of ER-negative cells, one would not expect to see such tumours going on to a partial response at 6 months as many achieve. The clinical results suggest that the change in ER expression is caused by a down-regulation mechanism.

These clinical findings that ER-positive tumours that become tamoxifen resistant do not lose ER expression are in keeping with the laboratory data described above and have implications for our understanding of acquired resistance to tamoxifen. While ER status predicts for sensitivity or insensitivity to first-line endocrine therapy, it appears to play little or no part in predicting or determining acquired resistance. This implies that the mechanisms of primary endocrine insensitivity and acquired (secondary) endocrine resistance are different. The former appears to be mediated via the ER (or lack of it), the latter not. The second point arising from the data and consistent with the point above is that these findings explain the clinical studies reporting that tamoxifen resistance does not necessarily mean complete endocrine resistance.

In a study of the synthetic progestogen, megace, 97 patients had tumours that initially responded or remained static on tamoxifen and then subsequently progressed, of whom 60 (62%) were reported to show a further period of response or static disease on second-line endocrine therapy, megace. In contrast, of 66 patients whose tumours progressed *de novo* on tamoxifen, only 17% showed an objective response or static disease on megace (Robertson *et al.*, 1989). Response to megace was better predicted by response to first-line tamoxifen than by tumour ER status. Similar response rates following tamoxifen therapy have been reported for second-line aromatase inhibitor therapy in postmenopausal patients (Smith *et al.*, 1981; Buzdar *et al.*, 1982; Harvey *et al.*, 1982; Kaye *et al.*, 1982; Murray and Pitt, 1982) and for oopherectomy in premenopausal patients (Margreiter and Wiegele, 1984; Sawka *et al.*, 1986).

Two clinical studies have been reported using the specific anti-oestrogen ICI 182,780. In the first study patients with primary operable (Stage I/II) breast cancer were treated with ICI 182,780 for 7 days between diagnosis and definitive surgery (DeFriend *et al.*, 1994). Patients were randomised to receive no treatment ($n = 19$), 6 mg of ICI 182,780 daily ($n = 21$) or 18 mg of ICI 182,780 daily ($n = 16$). Tumour specimens were available before randomisation to either no treatment or to ICI 182,780 and from the resected tumour at definitive surgery. There was down-regulation of ER on ICI 182,780 both at the 6 mg and the 18 mg dose. At the higher dose of ICI 182,780 (18 mg day$^{-1}$) five out of ten tumours showed absence of ER expression immunocytochemically in the primary tumour after 7 days' treatment (Nicholson *et al.*, 1994). The majority of ICI 182,780-treated tumours therefore continued to express ER, although at reduced levels. In the five tumours that did not express ER, it is much more likely that after such short-term treatment the absence of ER expression is as a result of down-regulation rather than true loss of ER. Down-regulation of ER can be induced *in vitro* by short-term treatment of ER-positive MCF-7 cells by pure anti-oestrogens (Nicholson *et al.*, 1994) without actual long-term loss of ER as already noted (Brunner *et al.*, 1993*a,b*; Clarke *et al.*, 1994). Similarly, down-regulation was also noted for oestrogen-inducible gene products PgR and pS2. These findings are qualitatively similar to those reported with tamoxifen except that reported down-regulation on tamoxifen was after 6 weeks. Nicholson reported that the fall in ER expression after 7 days on 18 mg of ICI 182,780 was greater

**Table I** Changes during tamoxifen therapy of ER expression in 33 tumours ER positive on pretreatment biopsy (repeat biopsy on tamoxifen *vs* pretreatment)

| | *Time (months) from pretreatment to repeat biopsy* <br> *<6 months* | | | *6 months* | | |
|---|---|---|---|---|---|---|
| UICC assessment at 6/12 | PR | SD | PD | PR | SD | PD |
| | 3 | 4 | 1 | 13 | 7 | 5 |
| Change in ER Expression | | | | | | |
| Down-regulated (but present) | 3 | 3 | – | 9 | 5 | 3 |
| No change | – | 1 | 1 | 4 | 2 | 2 |
| Up-regulated | – | – | – | – | – | – |

PR, partial response; SD, static disease; PD, progressive disease.

than after tamoxifen therapy. The tamoxifen-treated tumours were essentially the same group of tumours reported by Clarke *et al.* (1993) when the median duration of tamoxifen was 21 days – there was no significant down-regulation of ER by that time. One explanation for the reported differences between tamoxifen and ICI 182,780 could be the markedly different affinity of the two compounds for ER. It may therefore take longer for tamoxifen, which binds less avidly to the ER, to induce down-regulation.

The second study with ICI 182,780 was a phase II study to assess therapeutic efficacy (Howell *et al.*, 1995). Patients who had previously received tamoxifen as adjuvant therapy or as initial therapy for advanced disease and deemed to have been tamoxifen responsive were entered into the study. A total of 13/19 (69%) showed objective response or static disease on ICI 182,780, 12/19 (63%) for more than 6 months' duration. These second-line response rates are in keeping with those referenced above for second-line megace or aminoglutethamide in post-menopausal patients or ovarian ablation as second-line in premenopausal patients. With the median duration of response not yet having been reached at 18 months (Howell *et al.*, 1995) early indications are that ICI 182,780 may produce a longer duration of response than megace or aminoglutethamide, with the possibility of a further response if megace or aminoglutethamide is subsequently introduced. However, this will require confirmation.

As detailed above there appears to be continued expression of ER during tamoxifen therapy in almost all ER-positive tumours, even at the time of tumour progression. ICI 182,780 is known to bind to ER and the phase II study results imply that in the 69% of patients whose tumours responded the ER mechanism is still functional. Two patients with locoregional recurrence had primary tumour tissue from the time of their original diagnosis and a biopsy of the recurrent tumour while in partial remission on ICI 182,780. In the tumours of both patients there was continuing ER expression after 20 months on ICI 182,780. In one patient 100% of tumour cells stained positive for ER in both the primary tumour and the local recurrence (H-scores 180 and 160 respectively). In both tumour biopsies 100% of tumour cells also expressed PgR. In the second patient 70% of tumour cells in the primary tumour stained positive for ER, while in the regional lymph node recurrence 65% of cells stained positive on ICI 182,780 (H-scores 80 and 85 respectively). PgR expression was only available on the primary tumour – 95% of tumour cells expressed PgR (H-score 185) (JFR Robertson, unpublished data).

Therefore whatever the mechanism of acquired resistance to tamoxifen it does not, in the majority of cases, involve loss of ER expression, or apparently of receptor functionality. Possible mechanisms of acquired tamoxifen resistance such as ER variants (Fuqua *et al.*, 1991) are not the subject of this paper. Neither is the question whether the tumours in the remaining 30% of patients who were tamoxifen sensitive but did not respond to second-line endocrine agents (e.g. ICI 182,780), still have a functioning ER. This would depend on possible differences in the mechanism of resistance between this 30% of tumours and the 70% that responded to both tamoxifen and ICI 182,780. Tumours that responded to ICI 182,780 and subsequently progressed indicate that resistance is also acquired to the specific anti-oestrogen. In the *in vivo* experiments most tumour xenografts eventually developed resistance to tamoxifen and to ICI 182,780. The clinical findings are in keeping with xenograft experiments, which also showed a functioning ER in the presence of acquired endocrine resistance. If other mechanisms are responsible for acquired resistance to tamoxifen this would explain why ER is still expressed, still functional and yet is not as good a predictor of response to second-line endocrine therapy compared with prior response to tamoxifen.

The hypothesis of this paper is that ER expression is stable in breast cancer cells. While expression of ER in tumour cells is stable the relative or absolute number of ER-positive or ER-negative cells may vary during the course of patients' disease depending on a variety of host–tumour interactions. However, the evidence which has accumulated suggests that any relative or absolute change in the number of ER-positive and ER-negative cells in a tumour is not as a result of individual ER-positive tumour cells losing ER. If the hypothesis is true there are important implications for treatment from chemoprevention to acquired endocrine resistance in advanced disease. Equally if the hypothesis is true attempts to develop laboratory models of endocrine resistance where ER-positive tumours become ER negative need to be re-evaluated.

The concept that in breast cancers ER positivity drifts to ER negativity as a natural process of selection and increasing malignancy can no longer be supported. An alternative hypothesis is that ER is a stable phenotype in human breast cancer and ER positive-cells do not lose anti-oestrogen sensitivity by becoming ER negative.

**Acknowledgements**
Drs Elston and Ellis, Consultant Pathologists, City Hospital, Nottingham, reported the ER results described in Table I. I am grateful to them for reporting the ER results on the two patients receiving ICI 182,780. I also acknowledge the secretarial help of Mrs A Brown.

**References**

ALLEGRA JC, BARLOCK A, HUFF KK AND LIPPMAN ME. (1980). Changes in multiple or sequential oestrogen receptor determinations in breast cancer. *Cancer*, **45**, 792–794.

ANDERSON EDC, FORREST APM, LEVACK PA, CHETTY U AND HAWKINS RA. (1989). Response to endocrine manipulation and oestrogen receptor concentration in large operable primary breast cancer. *Br. J. Cancer*, **60**, 223–226.

BISHOP HM. (1982). Oestrogen receptors in human breast cancer. DM Thesis, University of Nottingham.

BOJAR, H. (1986). Quality control requirements in estrogen receptor determination. *Cancer Res.*, **46**, 4249(s)–4250(s).

BRENNER SE, CLARK, GM AND MCGUIRE, WL. (1988). Review: steroid receptors, cellular kinetics and lymph nodes status as prognostic factors in breast cancer. *Am. J. Med. Sci.*, **296**, 59–66.

BROOKS SC, SAUNDERS DE, SINGHAKOWINTA A AND VAITKEVICIUS VK. (1980). Relation of tumour content of estrogen and progesterone receptors with response of patient to endocrine therapy. *Cancer*, **46**, 2775–2778.

BRUNNER N, BOULAY V, FOJO A, FRETER CE, LIPPMAN ME AND CLARKE R. (1993*a*). Acquisition of hormone independent growth in MCF-7 cells is accompanied by increased expression of estrogen regulated genes but without detectable DNA amplifications. *Cancer Res.*, **53**, 283–290.

BRUNNER N, FRANDSEN TL, HOLST-HANSEN C, BEI M, THOMPSON EW, WAKELING AE, LIPPMAN ME AND CLARKE R. (1993*b*). MCF-7/LCC2 : a 4-hydroxytamoxifen resistant breast cancer variant which retains sensitivity to the steroid antiestrogen ICI 182,780. *Cancer Res.*, **53**, 3229–3232.

BUR ME, ZIMAROWSKI MJ, SCHIFF SJ, BAKER S AND LEW R. (1992). Estrogen receptor immunocytochemistry in carcinoma *in situ* of the breast. *Cancer*, **69**, 1174–1181.

BUZDAR AV, POWELL KC AND BLUMENSCHEIN GR. (1982). Aminoglutethamide after Tamoxifen in advanced breast cancer: MD Anderson Hospital experience. *Cancer Res.*, **42**, 3448s–3450s.

CAMPBELL FC, BLAMEY RW, ELSTON CW, MORRIS AH, NICHOLSON RI, GRIFFITHS K AND HAYBITTLE JL. (1981). Quantitative oestradiol receptor values in primary breast cancer and response of metastases to endocrine therapy. *Lancet*, **2**, 1317–1319.

CLARKE GM AND MCGUIRE WL. (1988). Steroid receptors and other prognostic factors in primary breast cancer. *Semin. Oncol.*, **15**, (no. 2 suppl). **1**, 20–25.

CLARKE RB, LAIDLAW IJ, JONES LJ, HOWELL A AND ANDERSON E. (1993). Effect of Tamoxifen on Ki67 labelling index in human breast tumours and its relationship to oestrogen and progesterone receptor status. *Br. J. Cancer*, **67**, 606–611.

CLARKE R, SKAAR T, BAUMANN IC, LEONESSA F, JAMES M, LIPPMAN J, THOMPSON EW, FRETER C AND BRUNNER N. (1994). Hormonal carcinogenesis in breast cancer: cellular and molecular studies of malignant progression. *Breast Cancer Res. Treat.*, **31**, 237–248.

COOKE T, GEORGE WD, SHIELD R, MAYNARD PV AND GRIFFITHS K. (1979). Oestrogen receptors and prognosis in early breast cancer. *Lancet*, **1**, 995–997.

COOMBES RC, POWLES TJ, BERGER V, WILSON P, MCCLELLAND RA, GAZET JC, TROTT PA AND FORD HT. (1987). Prediction of endocrine response in breast cancer by immunocytochemical detection of oestrogen receptor in fine-needle aspirate. *Lancet*, **2**, 701–703.

CROTON R, COOKE T, HOLT S, GEORGE WD, NICHOLSON RI AND GRIFFITHS K. (1981). Oestrogen receptors and survival in early breast cancer. *Br. Med. J.*, **283**, 1289–1291.

DALY RJ AND DARBRE PD. (1990). Cellular and molecular events in loss of estrogen sensitivity in ZR-75-1 and T47D human breast cancer cells. *Cancer Res.*, **50**, 5868–5875.

DAVIS BW, ZAVA DT, LOCHER GW, GOLDHIRSCH A AND HARTMANN WH. (1984). Receptor heterogeneity of human breast cancer as measured by multiple intratumoral assays of estrogen and progesterone receptor. *Eur. J. Cancer Clin. Oncol.*, **20**, 375–382.

DEFRIEND DJ, HOWELL A, NICHOLSON RI, ANDERSON E, DOWSETT M, MANSEL RE, BLAMEY RW, BUNDRED NJ, ROBERTSON JFR, SAUNDERS C, BAUM M, WALTON P, SUTCLIFFE F AND WAKELING A. (1994). Investigation of a new pure anti-estrogen (ICI 182,780) in women with primary breast cancer. *Cancer Res.*, **54**, 408–414.

ENCARNACION CA, CIOCCA DR, MCGUIRE WL, CLARK GM, FUGUA SAW AND OSBORNE CK. (1993). Measurement of steroid hormone receptors in breast cancer patients on Tamoxifen. *Breast Cancer Res. Treat.*, **26**, 237–246.

EUSELI V, CERASOLI PT, GUIDELLI-GUIDI S, GRILLI S, BUSSOLATI G AND AZZOPARDI JG. (1981). A two stage immunocytochemical method for oestrogen receptor analysis: correlation with morphologic parameters of breast carcinomas. *Tumori*, **67**, 315–323.

EWING TE, MURPHY LJ, NG M-L, PANG GYN, LEE CSL, WATTS CKJW AND SUTHERLAND RL. (1989). Regulation of epidermal growth factor receptor by progestins and glucocorticoids in human breast cancer cell lines. *Int. J. Cancer*, **44**, 744–752.

FABIAN C, STERNSON L, EL-SERAFI M, CAIN L AND HEARNE E. (1981). Clinical pharmacology of Tamoxifen in patients with breast cancer: correlation with clinical data. *Cancer*, **48**, 876–882.

FUQUA SA, FITZGERALD SD, CHARMNESS GC, TANDON AK, MCDONNELL DP, NAWAZ Z, O'MALLEY BW AND MCGUIRE WL. (1991). Variant human breast tumour estrogen receptor with constitutive transcriptional activity. *Cancer Res.*, **51**, 105–109.

GASKELL DJ, HAWKINS RA, SANGSTER K, CHETTY U AND FORREST APM. (1989). Relation between immunocytochemical estimation of oestrogen receptor in elderly patients with primary breast cancer and response to tamoxifen. *Lancet*, **1**, 1044–1046.

GIRI DD, DUNDAS SAC AND NOTTINGHAM JF AND UNDERWOOD JCE. (1989). Oestrogen receptors in benign epithelial lesions and intraduct carcinomas of the breast: an immunohistological study. *Histopathology*, **15**, 575–584.

GOTTARDIS MM AND JORDAN VC. (1988). Development of Tamoxifen-stimulated growth of MCF-7 tumours in athymic mice after long-term anti-estrogen administration. *Cancer Res.*, **48**, 5183–5187.

GOTTARDIS MM, JIANG S-Y, JENG M-H AND JORDAN VC. (1989). Inhibition of Tamoxifen-stimulated growth of an MCF-7 tumour variant in athymic mice by novel steroidal anti-estrogens. *Cancer Res.*, **49**, 4090–4093.

GREEN GL AND JENSEN EV. (1992). Monoclonal antibodies as probes for estrogen receptor detection and characteristics. *J. Steroid Biochem.*, **16**, 353–359.

HAHNEL R, WOODINGS T AND VIVIAN AB. (1979). Prognostic value of oestrogen receptors in primary breast cancer. *Cancer*, **44**, 671–675.

HAHNEL R AND TWADDLE E. (1985). The relationship between estrogen receptors in primary and secondary breast carcinomas and in sequential primary breast carcinomas. *Breast Cancer Res. Treat.*, **5**, 155–163.

HARLAND RNL, BARNES DM, HOWELL A, RIBEIRO G, TAYLOR J AND SELLWOOD RA. (1983). Variation in receptor status in cancer of the breast. *Br. J. Cancer*, **47**, 511–515.

HARVEY HA, LIPTON A, WHITE DS, SANTEN RJ, BORCHER AE, SHAFIK AS AND DIXON RJ. (1982). Cross-over comparison of Tamoxifen and Aminoglutethamide in advanced breast cancer. *Cancer Res.*, **42**, 3451s–3453s.

HAWKINS RA, HILL A AND FREEDMAN B. (1975). A simple method for the determination of estrogen receptor concentrations in breast tumours and other tumours. *Clin. Chim. Acta.*, **64**, 203–210.

HAWKINS RA, HILL A, FREEDMAN B, GORE SM, ROBERT MM AND FORREST APM. (1977). Reproducibility of measurements of oestrogen receptor concentration in breast cancer. *Br. J. Cancer*, **36**, 355–361.

HAWKINS RA, WHITE G, BUNDRED NJ, DIXON JM, MILLER WR, STEWART HJ AND FORREST ADM. (1987*a*). Prognostic significance of oestrogen and progesterone receptor activities in breast cancer. *Br. J. Surg.*, **74**, 1009–1013.

HAWKINS RA, SANGSTER K AND TESDALE AL, FERGUSON WA, KRAJEWSKI A, LEVAK PA AND FORREST P. (1987*b*). Experience with new assays for oestrogen receptors using monoclonal antibodies. *Biochem. Soc. Trans.*, **15**, 949–950.

HAWKINS RA, SANGSTER K AND TESDALE A. (1988). The cytochemical detection of oestrogen receptors in fine needle aspirates of breast cancer; correlation with biochemical assay and prediction of response to endocrine therapy. *Br. J. Cancer*, **58**, 77–80.

HOWELL A, BARNES DM, HARLAND RNL, REDFORD J, BRAMWELL VHC, WILKINSON MJS, SWINDELL R, CROWTHER D AND SELLWOOD RA. (1984). Steroid-hormone receptors in survival after first relapse in breast cancer. *Lancet*, **1**, 588–591.

HOWELL A, ANDERSON E, LAIDLAW I, SCHOR A, SCHOR S AND POTTEN C. (1994). Cyclical activity and 'ageing' of the human breast: clues to assessment of risk and strategies for prevention. In: *Endocrine Therapy of Breast Cancer VI*, Howell A (ed.) pp. 27–46. Springer: Berlin.

HOWELL A, DEFRIEND D, ROBERTSON J, BLAMEY R AND WALTON P. (1995). Response to a specific anti-oestrogen (ICI 182,780) in Tamoxifen resistant breast cancer. *Lancet*, **345**, 29–30.

JOHNSTON SRD, HAYNES BP, SMITH IE, JARMAN M, SACKS NPM, EBBS SR AND DOWSETT M. (1994). Acquired tamoxifen resistance in human breast cancer and reduced intra-tumoral drug concentration. *Lancet*, **2**, 1521–1522.

JONAT W, MAASS H AND STEGNER HE. (1986). Immunocytochemical measurement of estrogen receptors in breast cancer tissue samples. *Cancer Res.*, **46**, 4296s–4298s.

JORDAN VC, JACOBSEN HI AND KEENAN EJ. (1986). Determination of estrogen receptor in breast cancer using monoclonal antibody technology. Results of a multi-center study in the United States. *Cancer Res.*, **46**, (suppl.) 4237–4240.

JORDAN CV. (1994). Drug resistance to antioestrogen therapy. In *Endocrine Therapy of Breast Cancer VI*, Howell A (ed.) pp. 61–68. Springer: Berlin.

KAYE SB, WOODS RL, FOX RM, COATES AS AND TATTERSALL MHN. (1982). Use of Aminoglutethamide as second-line endocrine therapy in metastatic breast cancer. *Cancer Res.*, **42**, 3445s–3447s.

KIANG DT AND KENNEDY BJ. (1977). Factors affecting estrogen receptors in breast cancer. *Cancer*, **40**, 1571–1576.

KING WJ AND GREEN GL. (1984). Monoclonal antibodies localize oestrogen receptor to the nucleus of target cells. *Nature*, **307**, 745–747.

KNIGHT WA, LIVINGSTON RB, GREGORY EJ AND MCGUIRE WL. (1977). Estrogen receptor as an independent prognostic factor for early recurrence in breast cancer. *Cancer Res.*, **37**, 4669–4671.

KOHLER G AND MILSTEIN C. (1975). Continuous culture of fused cells secreting antibody of predefined specificity. *Nature*, **256**, 494–497.

LECLERQ G, BOJAR H, GOUSSARD J, NICHOLSON RI, PICHON M-F, PIFFANELLI A, POVSETTE A, THORPE S AND LONSDORFER M. (1986). Abbot monoclonal enzyme immunoassay measurement of estrogen receptors in human breast cancer. A European multicenter study. *Cancer Res.*, **46**, (suppl.) 4233–4236.

LIPPMAN ME AND ALLEGRA JC. (1980). Quantitative estrogen receptor analyses. The response to endocrine and cytotoxic chemotherapy in human breast cancer and the disease-free interval. *Cancer*, **46**, 2829–2834.

MCCARTY KS JR, SZABO E, FLOWERS JL, COX EB, LEIGHT GS, MILLER L, KAURATH J, SOPER JT, BUDWIT DA, GREASMAN WT, SEIGLER HF AND MCCARTY KS SR. (1986). Use of a monoclonal anti-estrogen receptor antibody in the immunohistochemical evaluation of human tumours. *Cancer Res.*, (suppl.) **46**, 4244(s)–4248(s).

MCCLELLAND RA, BERGER U, MILLER LS, POWLES TJ AND COOMBES RC. (1986*a*). Immunocytochemical assay for estrogen receptor in patients with breast cancer: relationship to a biochemical assay and to outcome of therapy. *J. Clin. Oncol.*, **4**, 1171–1176.

McCLELLAND RA, BERGER U, MILLER LS, POWLES TJ, JENSEN EV AND COOMBES RC. (1986*b*). Immunocytochemical assay for estrogen receptor: relationship to outcome of therapy in patients with advanced breast cancer. *Cancer Res.*, **46**, 4241(s)–4243(s).

McCLELLAND RA, FINLAY P, DIXON AR, ROBERTSON JFR, ELLIS IO, BLAMEY RW AND NICHOLSON RI. (1993). Epidermal growth factor receptors and oestrogen receptor expression in breast cancer: Relationship to endocrine sensitivity. *Oncol. (Life Sci. Adv.)*, **12**, 143–155.

McGUIRE WL, CARBONNE PP, SEARS ME AND ESCHER GC. (1975). Estrogen receptors in human breast cancer: an overview. In *Estrogen Receptors in Human Breast Cancer*, McGuire WL, Carbonne PP and Vollmer EP (eds.) pp. 1–7. Raven Press: New York.

McGUIRE WL, ZARA DT, HORWITZ KB AND CHAMNESS GC. (1978). Hormones, receptors and breast cancer. In *Proceedings of the Sixth Tenovus Workshop on Tumour Markers*, Griffiths K, Neville AM and Pierrepoint CG (eds.) pp. 153–161. Alpha Omega Publishing: Cardiff.

MALAFA M, CHAUDHURI B, THOMFORD NR AND CHAUDHURI PK. (1990). Estrogen receptors in ductal carcinoma in situ of breast. *Ann. Surg.*, **56**, 436–439.

MARGREITER R AND WIEGELE J. (1984). Tamoxifen (Nolvadex) for premenopausal patients with advanced breast cancer. *Breast Cancer Res. Treat.*, **4**, 45–48.

MARKOPOULOS C, BERGER U, WILSON P, GAZET JC AND COOMBES RC. (1988). Oestrogen receptor content of normal breast cells and breast carcinoma throughout the menstrual cycle. *Br. Med. J.*, **296**, 1349–1351.

MASON BH, HOLDAWAY IM, MULLINS PR, YEE LH AND KAY RG. (1983). Progesterone and estrogen receptors as prognostic variables in breast cancer. *Cancer Res.*, **43**, 2985–2990.

MASTERS JRW, HAWKINS RA, SANGSTER K, HAWKINS W, SMITH II, SHIVAS AA, ROBERTS MM AND FORREST APM. (1978). Oestrogen receptors, cellularity, elastoses and menstrual status in human breast cancer. *Eur. J. Cancer*, **14**, 303–307.

MAYNARD PV, BLAMEY RW, ELSTON CW, HAYBITTLE JL AND GRIFFITHS K. (1978). Estrogen receptor assay in primary breast cancer and early recurrence of the disease. *Cancer Res.*, **38**, 4292–4295.

MOOLGAVAKAR SH, DAY NE AND STEVENS RG. (1980). Two stage model for carcinogenesis: epidemiology of breast cancer in females. *J. Natl Cancer Inst.*, **65**, 559–569.

MORROW M AND JORDAN VC. (1993). Molecular mechanisms of resistance to Tamoxifen therapy in breast cancer. *Arch. Surg.*, **128**, 1187–1191.

MULLICK A AND CHAMBON P. (1990). Characterisation of the estrogen receptor in two antioestrogen-resistant cell lines, LY2 and T47D. *Cancer Res.*, **50**, 333–338.

MURPHY CS, PINK JJ AND JORDAN VC. (1990). Characteristics of a receptor-negative hormone non-responsive clone derived from T47D human breast cancer cell line kept under oestrogen free conditions. *Cancer Res.*, **50**, 7285–7292.

MURPHY C, SIBBERING M, ELLIS IO, PINDER S, BELL JA, BLAMEY RW AND ROBERTSON JFR. Progesterone receptor status in DCIS (submitted).

MURRAY PA, GOMM J, RICKETTS D, POWLES T AND COOMBES RC. (1994). The effect of endocrine therapy on the levels of oestrogen and progesterone receptor and transforming growth factor-$\beta$1 in metastatic human breast cancer: an immunocytochemical study. *Eur. J. Cancer*, **2**, 1218–1222.

MURRAY RML AND PITT P. (1982). Aminoglutethamide in Tamoxifen-resistant patients: The Melbourne experience. *Cancer Res.*, **42**, 3437s–3441s.

NAMER M, LALAANE C AND BAULIEU E-E. (1980). Increase of progesterone receptor by Tamoxifen as a hormonal challenge test in breast cancer. *Cancer Res.*, **40**, 1750–1752.

NEWSOME JF, AVIS FP, HAMMOND JE AND SHERWOOD S. (1981). Sampling procedures in estrogen receptor determinations. *Ann. Surg.*, **193**, 549–554.

NICHOLSON RI, COLIN P, FRANCIS AB, KESHRA R, FINLAY P, WILLIAMS M, ELSTON CW, BLAMEY RW AND GRIFFITHS K. (1986). Evaluation of an enzyme immunoassay for oestrogen receptors in human breast cancers. *Cancer Res.*, **46**, (suppl.) 4299s–4230s.

NICHOLSON RI, BOUZUBAR N, WALKER KJ, McCLELLAND R, DIXON AR, ROBERTSON JFR, ELLIS IO AND BLAMEY RW. (1991). Hormone sensitivity in breast cancer: influence of heterogeneity of oestrogen receptor expression and cell proliferation. *Eur. J. Cancer*, **27**, 908–913.

NICHOLSON RI, GEE JMW, EATON CL, MANNING DL, MANSEL RE, SHARMA AK, DOUGLAS-JONES A, PRICE-THOMAS M, HOWELL A, DeFRIEND DJ, BUNDRED NJ, ANDERSON E, ROBERTSON JFR, BLAMEY RW, DOWSETT M, BAUM M, WALTON P AND WAKELING AE. (1994). Pure antioestrogens in breast cancer: experimental and clinical observations. In *Sex Hormones and Anti-hormones in Endocrine-dependent Pathologies*, Motta M and Serio M (eds.) pp. 347–360. Excerpta Medica: Amsterdam.

NOGUCHI S, MIYAUDI K, NISHIZAWA Y AND KOYAMA H. (1988). Induction of progesterone receptor with Tamoxifen in human breast cancer with special reference to its behaviour over time. *Cancer*, **61**, 1345–1349.

NOMURA Y, TASHIRO H AND SHINOZUKA K. (1985). Changes in steroid hormone receptor content by chemotherapy and/or endocrine therapy in advanced breast cancer. *Cancer*, **55**, 546–551.

OSBORNE CK. (1993). Mechanisms for tamoxifen resistance in breast cancer: possible role of tamoxifen metabolism. *J. Steroid. Biochem. Mol. Biol.*, **47**, 83–89.

OSBORNE CK, YOCHMOWITZ MG, KNIGHT WA AND McGUIRE WL. (1980). The value of oestrogen and progesterone receptors in the treatment of breast cancer. *Cancer*, **46**, 2884–2888.

OSBORNE CK, CORONADO EB AND ROBINSON JP. (1987). Human breast cancer in the athymic nude mouse: cytostatic effects of long-term antiestrogen therapy. *Eur. J. Cancer Clin. Oncol.*, **23**, 188–196.

OSBORNE CK, CORONADO EB, ALLRED DC, WIEBE V AND DE GREGORIO M. (1991). Acquired tamoxifen resistance: correlation with reduced tumour levels of tamoxifen and isomerisation of trans-4-hydroxytamoxifen. *J. Natl Cancer Inst.*, **83**, 1477–1482.

OSBORNE CK, JARMAN M, MACAGUE R, CORONADO EB, DE GREGORIO MW, HILSENBECK SG AND WAKELING AE. (1994). The importance of tamoxifen metabolism in tamoxifen-stimulated breast tumour growth. *Cancer Chemother. Pharmacol.*, **34**, 89–95.

PAIK S, HARTMANN DP, DICKSON RB AND LIPPMAN ME. (1994). Antiestrogen resistance in ER positive breast cancer cells. *Breast Cancer Res. Treat.*, **31**, 301–307.

PALLIS L, WILKING N, CEDERMARK B, RUTQUISK LE AND SKOOG L. (1992). Receptors for estrogen and progesterone in breast carcinoma *in situ*. *Anticancer Res.*, **12**, 2113–2115.

PARIDAENS R, SYLVESTER RJ, FERAZZI E, LEGROS N, LECLERCQ G AND HEUSON JC. (1980). Clinical significance of the quantitative assessment of estrogen receptors in advanced breast cancer. *Cancer*, **46**, 2889–2895.

PEETZ ME, NUNLEY DL, MOSELEY HS, KEENAN EJ, DAVENPORT CED AND FLETCHER WS. (1982). Multiple simultaneous and sequential estrogen receptor values in patients with breast cancer. *Am. J. Surg.*, **143**, 591–594.

POLLER DN, SNEAD DRJ, ROBERTS EC, GALEA M, BELL JA, GILMOUR A, ELSTON CW, BLAMEY RW AND ELLIS IO. (1993). Oestrogen receptor expression in ductal carcinoma in situ of the breast: relationship to flow cytometric analysis of DNA and expression of the C-erbB2 oncoprotein. *Br. J. Cancer*, **68**, 156–161.

ROBERTS AB, FROLIK CA, ANGANO MA AND SPORN MB. (1983). Transforming growth factors from neoplastic and non-neoplastic tissues. *Fed. Proc.*, **42**, 2621–2626.

ROBERTS MM, RUBENS RD, KING RJB, HAWKINS RA, MILLS RR, HAYWARD JL AND FORREST APM. (1978). Oestrogen receptors and response to endocrine therapy in advanced breast cancer. *Br. J. Cancer*, **38**, 431–437.

ROBERTSON JFR, WILLIAMS MR, TODD J, NICHOLSON RI, MORGAN DAL AND BLAMEY RW. (1989). Factors predicting the response of patients with advanced breast cancer to endocrine (Megace) therapy. *Eur. J. Cancer Clin. Oncol.*, **25**, 469–475.

ROBERTSON JFR, ELLIS IO, NICHOLSON RI, ROBINS A, BELL J AND BLAMEY RW. (1991). Cellular effects of tamoxifen in primary breast cancer. *Breast Cancer Res. Treat.*, **20**, 117–123.

ROBERTSON JFR, BATES K, PEARSON D, BLAMEY RW AND NICHOLSON RI. (1992). Comparison of two oestrogen receptor assays in the prediction of the clinical course of patients with advanced breast cancer. *Br. J. Cancer*, **65**, 727–730.

SAINSBURY JRC, FARNDON JR, SHERBET GV AND HARRIS AL. (1985). Epidermal growth factor receptors and oestrogen receptors in human breast cancer. *Lancet*, **1**, 364–366.

SAMAAN NA, BUZDAR AV, ALDINGER KA, SCHULTZ PN, YANG K, ROMSDAHL MM AND MARTIN R. (1981). Estrogen receptor - a prognostic factor in breast cancer. *Cancer*, **47**, 554–560.

SAWKA CA, PRITCHARD PI, PATERSON AHG, SUTHERLAND DJA, THOMSON DB, SHELLEY WE, MYERS RE, MOBBS BG, MALKIN A AND MEAKIN JW. (1986). Role and mechanism of action of Tamoxifen in premenopausal women. *Cancer Res.*, **46,** 3152–3156.

SHARMA AK, HORGAN K, DOUGLAS-JONES A, MCCLELLAND R, GEE J AND NICHOLSON RI. (1994). Dual immunocytochemical analysis of oestrogen and epidermal growth factor receptors in human breast cancer. *Br. J. Cancer*, **69,** 1032–1037.

SILFVERSWARD C, SKOOG L, HUMLS S, GUSTAFSSON SA AND NORDENSKOJD B. (1980). Intratumoral variation of cytoplasmic and nuclear estrogen receptor concentrations in human mammary carcinoma. *Eur. J. Cancer*, **16,** 59–65.

SMITH IE, HARRIS AL, MORGAN M, FORD HT, GAZET J-C, HARMER C, WHITE H, PARSONS CA, VITTARDO A, WALSH G AND MCKINNA JA. (1981). Tamoxifen versus aminoglutethamide in advanced breast cancer: a randomised crossover trial. *Br. J. Med.*, **283,** 1432–1434.

SOOMRO S, SHOUSHA S AND SINNETT HD. (1992). Oestrogen and progesterone receptors in screen-detected breast carcinoma. An immunohistological study using paraffin sections. *Histopathology*, **21,** 543–547.

STRAUS MJ, MORAN RE, MULLER RE AND WAITZ HH. (1982). Estrogen receptor heterogeneity and the relationship between estrogen receptor and the tritiated thymidine labelling index in human breast cancer. *Oncology*, **39,** 197–200.

TAYLOR RE, POWLES TJ, HUMPHREYS J, BETHLEHEIM R, DOWSETT M, CASEY AJ, NEVILLE AM AND COOMBES RR. (1982). Effects of endocrine therapy on steroid receptor content of breast cancer. *Br. J. Cancer*, **45,** 80.

THORPE SM. (1987). Monoclonal antibody technique for detection of estrogen receptors in human breast cancer. Greater sensitivity and more accurate classification of receptor status than dextran-coated charcoal method. *Cancer Res.*, **47,** 6572–6575.

TILLEY WD, KEIGHTLEY DD AND CANT EL. (1978). Inter-site variation of estrogen receptors in human breast cancer. *Br. J. Cancer*, **38,** 544–546.

TOI M, HAMADA Y, NAKAMURA T, MUKAIDA H, SUEHIRO S, WADA T, TOGE T, NIIMOTO M AND HATTORI T. (1989). Immunocytochemical and biochemical analysis of epidermal growth factor expression in human breast cancer tissues: Relationship to oestrogen receptor and lymphatic invasion. *Int. J. Cancer*, **43,** 220–225.

VAN DEN BERG HW, LYNCH M, MARTIN J, NELSON J, DICKSON GR AND CROCKARD AD. (1989). Characterisations of a Tamoxifen-resistant variant of the ZR-75-1 human breast cancer cell line (ZR-95-9a1) and stability of the resistant phenotype. *Br. J. Cancer*, **59,** 522–526.

WALKER KJ, BOUZABAR N, ROBERTSON J, ELLIS IO, ELSTON CW, BLAMEY RW, WILSON DW, GFIFFITHS K AND NICHOLSON RI. (1988). Immunocytochemical localisation of estrogen receptor in human breast tissue. *Cancer Res.*, **48,** 6517–6522.

WALKER KJ, MCCLELLAND RA, CANDLISH W AND NICHOLSON RI. (1992). Heterogeneity of oestrogen receptor expression in normal and malignant breast tissue. *Eur. J. Cancer*, **28,** 34–37.

WALT AJG, SINGHAKOWINTA A, BROOKS SC AND CORTEZ A. (1976). The surgical implications of estrophile protein estimations in carcinoma of the breast. *Surgery*, **80,** 506–512.

WASEDA N, KATO Y, IMURA H AND KURATA M. (1981). Effects of Tamoxifen on estrogen and progesterone receptors in human breast cancer. *Cancer Res.*, **41,** 1984–1988.

WILBUR DC AND BARROWS GH. (1993). Estrogen and progesterone receptor and cerbB2 oncoprotein analysis in pure in situ breast carcinoma: an immunohistochemical study. *Mod. Pathol.*, **6,** 114–120.

WILKINSON P, RIBEIRO G, ADAM H AND PATTERSON J. (1980). Clinical pharmacology of Tamoxifen and N-desmethyl Tamoxifen in patients with advanced breast cancer. *Cancer Chemother. Pharmacol.*, **5,** 109.

WILLIAMS MR, TODD JH, ELLIS IO, DOWLE CS, HAYBITTLE JL, ELSTON CW, NICHOLSON RI, GRIFFITHS K AND BLAMEY RW. (1987). Oestrogen receptors in primary and advanced breast cancer: An eight year review of 704 cases. *Br. J. Cancer*, **55,** 67–73.

YANO T, KORKOT E, PINSKI J, SZEPESHAZI IC, MILOVANOVIC S, GROOT K, CLARKE R, COMARU-SCHALLY AM AND SCHALLY AV. (1992). Inhibition of growth of MCF-6 - MIII human breast carcinoma in nude mice by treatment with agonists or antagonists of LH-RH. *Br. Cancer Res. Treat.*, **21,** 35–45.